# Supplementary material for: Egalitarian preferences in young children depend on the genders of the interacting partners
Source: Commun Psychol. 2024 Sep 25;2:89. doi: 10.1038/s44271-024-00139-9 (PMC11424646; doi:10.1038/s44271-024-00139-9)
Supplement: Supplementary file 2 — Supplementary Materials [file 44271_2024_139_MOESM2_ESM.pdf]

1 Bootstrapped choice data per group

Table S1-4 shows the percentages of inequity rejection (median ± standard deviation [std], of the bootstrapped distributions of choices) underlying Figure 2 in the main manuscript.

Table S1-4 Bootstraped choices (distributin median ± std) for each subgroup per dilemma. F: female; M: male, FA: female allocator; MA: male allocator; FR: female recipient; MR: male recipient

S1 - DI Non-Costly<br>Age Group		Gender	median±std	Dyad	median±std
3-4 year	0.57±0.03	F: allocator	0.63±0.05	FA-FR	0.53±0.05
		F: recipient	0.45±0.05	FA-MR	0.71±0.05
		M: allocator	0.52±0.05	MA-FR	0.36±0.05
		M: recipient	0.67±0.05	MA-MR	0.63±0.05
5-6 year	0.72±0.03	F: allocator	0.68±0.04	FA-FR	0.57±0.05
		F: recipient	0.62±0.05	FA-MR	0.84±0.03
		M: allocator	0.77±0.04	MA-FR	0.68±0.05
		M: recipient	0.85±0.03	MA-MR	0.86±0.03
7-8 year	0.91±0.02	F: allocator	0.88±0.03	FA-FR	0.89±0.03
		F: recipient	0.93±0.03	FA-MR	0.89±0.02
		M: allocator	0.96±0.02	MA-FR	0.96±0.02
		M: recipient	0.9±0.03	MA-MR	0.94±0.02

S2 - DI Costly<br>Age Group		Gender	median±std	Dyad	median±std
3-4 year	0.31±0.03	F: allocator	0.31±0.05	FA-FR	0.2±0.04
		F: recipient	0.21±0.04	FA-MR	0.41±0.05
		M: allocator	0.3±0.04	MA-FR	0.21±0.04
		M: recipient	0.39±0.05	MA-MR	0.37±0.05
5-6 year	0.55±0.03	F: allocator	0.45±0.04	FA-FR	0.42±0.04
		F: recipient	0.49±0.04	FA-MR	0.5±0.03
		M: allocator	0.64±0.05	MA-FR	0.57±0.05
		M: recipient	0.61±0.04	MA-MR	0.71±0.05
7-8 year	0.78±0.03	F: allocator	0.77±0.04	FA-FR	0.7±0.05
		F: recipient	0.76±0.04	FA-MR	0.85±0.03
		M: allocator	0.8±0.04	MA-FR	0.81±0.04
		M: recipient	0.81±0.04	MA-MR	0.77±0.04

S3 - AI Non-Costly Age Group		Gender	median±std	Dyad	median±std
3-4 year	0.57±0.03	F: allocator	0.72±0.05	FA-FR	0.87±0.03
		F: recipient	0.66±0.05	FA-MR	0.59±0.05
		M: allocator	0.43±0.05	MA-FR	0.43±0.05
		M: recipient	0.5±0.05	MA-MR	0.42±0.05
5-6 year	0.5±0.03	F: allocator	0.46±0.05	FA-FR	0.54±0.05
		F: recipient	0.59±0.05	FA-MR	0.34±0.04
		M: allocator	0.54±0.05	MA-FR	0.65±0.05
		M: recipient	0.4±0.05	MA-MR	0.44±0.05
7-8 year	0.68±0.03	F: allocator	0.73±0.04	FA-FR	0.85±0.04
		F: recipient	0.72±0.05	FA-MR	0.66±0.04
		M: allocator	0.62±0.05	MA-FR	0.61±0.04
		M: recipient	0.64±0.04	MA-MR	0.65±0.05

S4 - AI Costly Age Group		Gender	median±std	Dyad	median±std
3-4 year	0.31±0.03	F: allocator	0.34±0.05	FA-FR	0.33±0.05
		F: recipient	0.31±0.05	FA-MR	0.35±0.05
		M: allocator	0.27±0.04	MA-FR	0.28±0.04
		M: recipient	0.3±0.05	MA-MR	0.26±0.04
5-6 year	0.31±0.03	F: allocator	0.37±0.05	FA-FR	0.35±0.05
		F: recipient	0.32±0.05	FA-MR	0.39±0.05
		M: allocator	0.25±0.04	MA-FR	0.29±0.04
		M: recipient	0.29±0.05	MA-MR	0.2±0.04
7-8 year	0.54±0.03	F: allocator	0.62±0.05	FA-FR	0.59±0.05
		F: recipient	0.57±0.05	FA-MR	0.63±0.04
		M: allocator	0.43±0.05	MA-FR	0.55±0.05
		M: recipient	0.49±0.05	MA-MR	0.23±0.04

2 Mixed-effects model split by Inequity or Cost type

Table S5 and S6 show the results for the mixed-effects model, separated by type of inequity (DI vs AI). Binary choices (1 per N=2 conditions, N=279 subjects per choice) are modelled within subjects, with Age as a between-subjects variable. Coefficients are shown in logits in the tables. For DI and for AI choices separately, the significant effect of Cost remains (higher rejections for non-costly unequal choice alternatives), and a significant effect of Age is apparent. In neither case does adding the interaction between CostType x Age improve the model fit (LRT_{DI}, $\chi^2_1 = 0.0354$, $p = 0.8507$; LRT_{AI}, $\chi^2_1 = 1.9173$, $p = 0.1662$)

Table S5 Mixed-Effects model for DI choices only. Binary choices (1 per condition, N=279 subjects) are modelled within subjects.

Baseline Model only for DI	Estimate	SE	Z-val	P-value
(Intercept)	-3.8241	0.6659	-5.743	9.32E-09
Non-Costly	1.0757	0.235	4.577	4.72E-06
Age (continuous)	0.6873	0.1082	6.351	2.13E-10

Table S6 Mixed-Effects model for AI choices only. Binary choices (1 per condition, N=279 subjects) are modelled within subjects.

Baseline Model only for AI	Estimate	SE	Z-val	P-value
(Intercept)	-2.56773	0.57043	-4.501	6.75E-06
Non-Costly	0.95522	0.21005	4.548	5.43E-06
Age (continuous)	0.32178	0.08483	3.793	1.49E-04

Table S7 and S8 show the results for the mixed-effects model, separated by type of cost (non-costly vs costly). Binary choices (1 per N=2 conditions, N=279 subjects per choice) are modelled within subjects, with Age as a between-subjects variable. Coefficients are shown in logits in the tables.

As in Tables S5-6, the coefficient for Age is highly significant. The interaction between InequityType and Age is significant for the non-costly choices, but not for the costly choices. This difference (that is, the interaction between CostType x InequityType x Age), however, itself does not reach significance (see main text).

Table S7 Mixed-Effects model for non-costly choices only. Binary choices (1 per condition, N=279 subjects) are modelled within subjects.

Baseline model only for non-costly choices	Estimate	SE	Z-val	P-value
(Intercept)	-2.4027	0.6475	-3.711	0.000207
Age (continuous)	0.588	0.1102	5.338	9.41E-08
AI over DI	1.5691	0.8477	1.851	0.064173
Age x AI	-0.4046	0.1397	-2.896	0.003775

Table S8 Mixed-Effects model for costly choices only. Binary choices (1 per condition, N=279 subjects) are modelled within subjects

Baseline model only for costly choices	Estimate	SE	Z-val	P-value
(Intercept)	-3.2128	0.6643	-4.837	1.32E-06
Age (continuous)	0.5785	0.107	5.408	6.37E-08
AI over DI	0.4036	0.8756	0.461	0.645
Age x AI	-0.2083	0.1371	-1.519	0.129

3 Analysis split on CostType

Figures S1-2 shows the predicted effects (using the "Effects" package[1–3]). To show the fits separately for costly and non-costly choices, an additional non-significant interaction of CostType:InequityType:Age was modelled in Fig.1. Below, the fits are shown when a priori splitting the choice data on CostType.

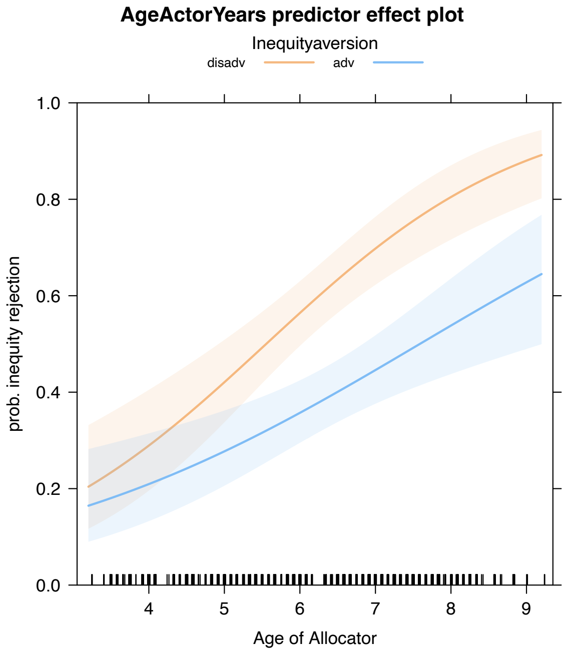

Figure S1 Predicted effect of InequityType by Age on inequity rejection, for costly choices only

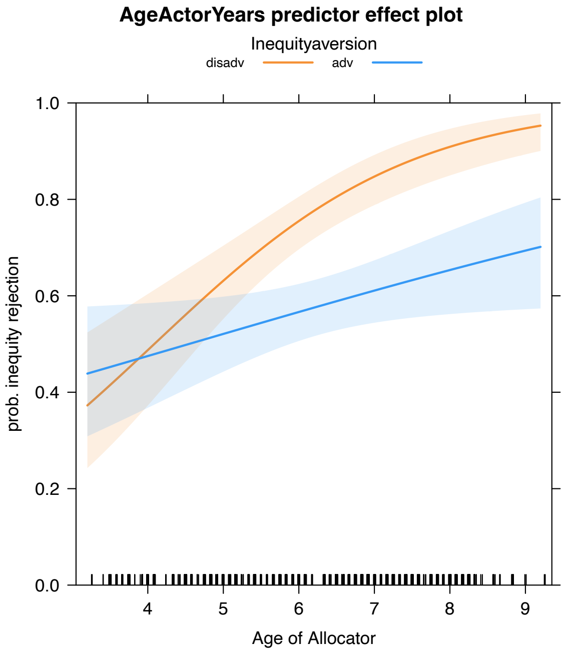

Figure S2 Predicted effect of InequityType by Age on inequity rejection, for non-costly choices only

4 Likelihood-ratio tests for Cost Type interactions

In the likelihood-ratio tests oulined below in Table S9 and S10, the hypothesis that adding some type of interaction with CostType would improved the models (for models including RecipientGender or AllocatorGender, respectively) is tested. None of the additional modeled interactions improve the model fit.

Table S9 Likelihood-ratio tests for models that explore the addition of interactions with Cost Type over the model that includes Recipient Gender

	npar	AIC	BIC	logLik	Chisq	Df	pval
Model including Recipient Gender	8	1346	1386.1	-664.99			
Addition of Cost Type x Inequity Type	9	1347.8	1392.9	-664.89	0.2028	1	0.6524
Addition of Cost Type x Age (continuous)	9	1347.3	1392.4	-664.64	0.7045	1	0.4013
Cost*Inequityaversion* GenderRecipient	11	1349.4	1404.6	-663.72	2.5346	3	0.4691

Table S10 Likelihood-ratio tests for models that explore the addition of interactions with Cost Type over the model that includes Allocator Gender

	npar	AIC	BIC	logLik	Chisq	Df	pval
Model including Recipient Gender	8	1353.1	1393.2	-668.55	1337.1		
Addition of Cost Type x Inequity Type	9	1355	1400.1	-668.49	1337	1	0.7145
Addition of Cost Type x Age (continuous)	9	1354.4	1399.6	-668.21	0.6928	1	0.4052
Cost*Inequityaversion* GenderAllocator	11	1358.1	1413.3	-668.05	1.0096	3	0.7989

5 Comparison raw choice data to Blake et al. (2015)

The data from Blake et al. (2015)[4] include repeated choices from children pertaining the acceptance or rejection of a given unequal (U) or equal (E) distribution. The authors collected choice data from 7 societies and published their raw data[5]. Participants went through a block of E and U choices (block order counterbalanced between participants), and DI vs AI was used as a between-subjects factor. Here we plot data from both experiments together (costly choices from our experiment, Canadian + US children from Blake). The raw choice data follow roughly the same pattern, with a strong increase in AI for FF pairs, but not MM pairs. Rejection rates in the AI condition in our data are higher (especially for girls), potentially due to the fact that a rejection led to the 1:1 outcome, instead of a zero payoff, and the absolute and relative payout difference was smaller than in Blake's experiment. In the DI condition, conversely, the 1:1 alternative in our experiment seemingly leads to overall higher DI rejection rates, even if this is costly to the Allocator (from 2 to 1).

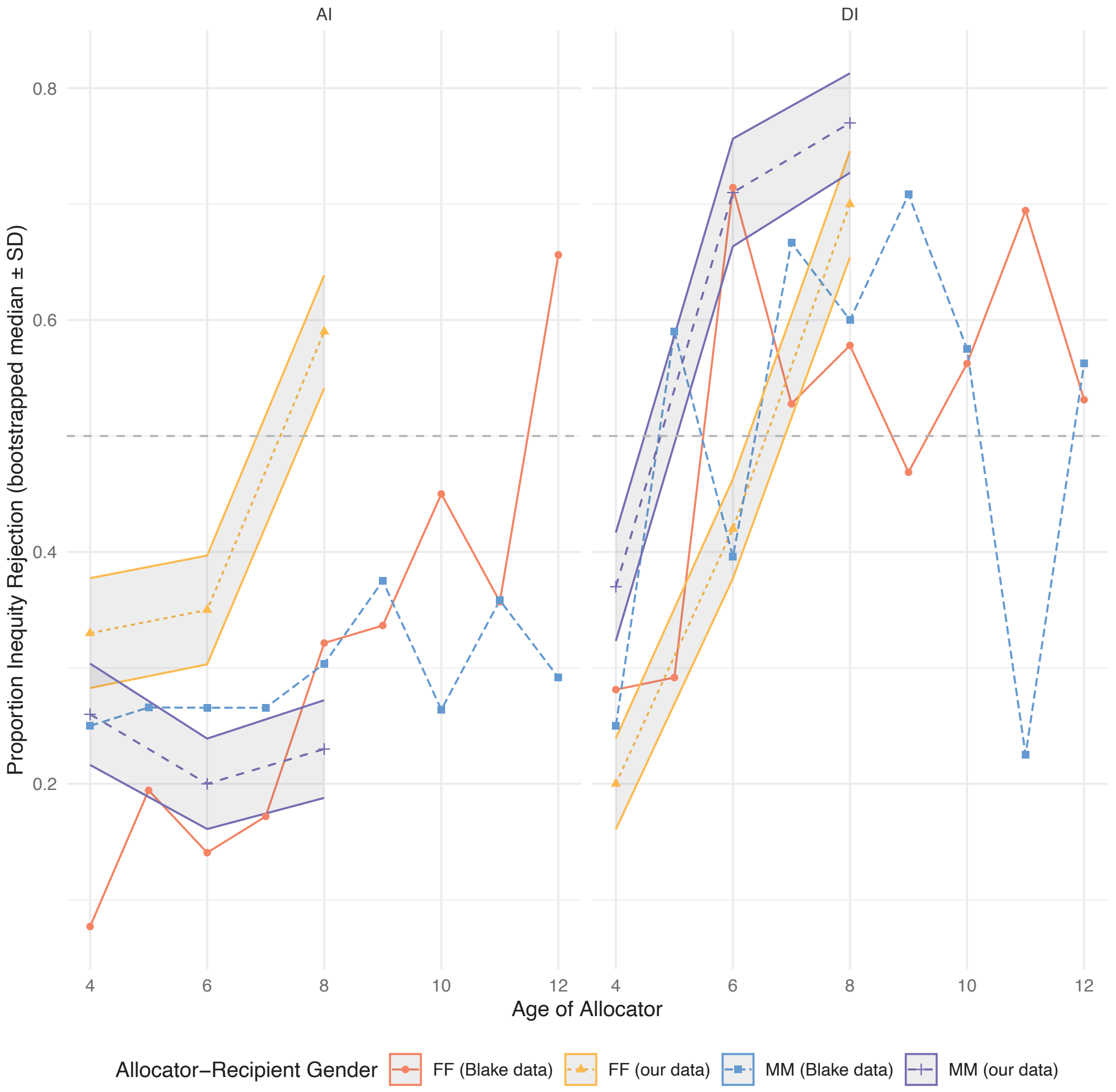

Figure S3 Bootstrapped median rejection rates (±std error bands) for our data and average rejection rates for data from Blake et al. 2015, separated for AI (left) and DI (right). Data from our three age groups is presented as 4,6, and 8yo, respectively. Our data: FF: Female Allocator with Female Recipient N=76; MM: Male Allocator with Male Recipient N=64.

6 Model Fits and Model Parameters for Re-analysis AI from Blake Dataset (US + Canada)

In this re-analysis from the Blake dataset, we specifically zoom in on the AI unequal distribution from the WEIRD (US+Canada) children, across their full age range. These model predictions in Fig. S4 show a similar trend to our data, and indeed, the coefficient for Age x GenderAllocator is significant (see Table S11).

Table S11 Mixed-Effects model for the unequal distributions in the Blake dataset. WEIRD children (US + Canadian), children in AI condition only.

AI Gender Effect	Estimate	SE	Z-val	P-value
(Intercept)	-4.95	0.83	-5.99	0.00000
Age (continuous)	0.44	0.10	4.57	0.00000
Male Allocators	3.27	1.18	2.78	0.00548
Trial Number	0.00	0.02	0.03	0.97341
Male Allocators x Age	-0.39	0.14	-2.82	0.00479

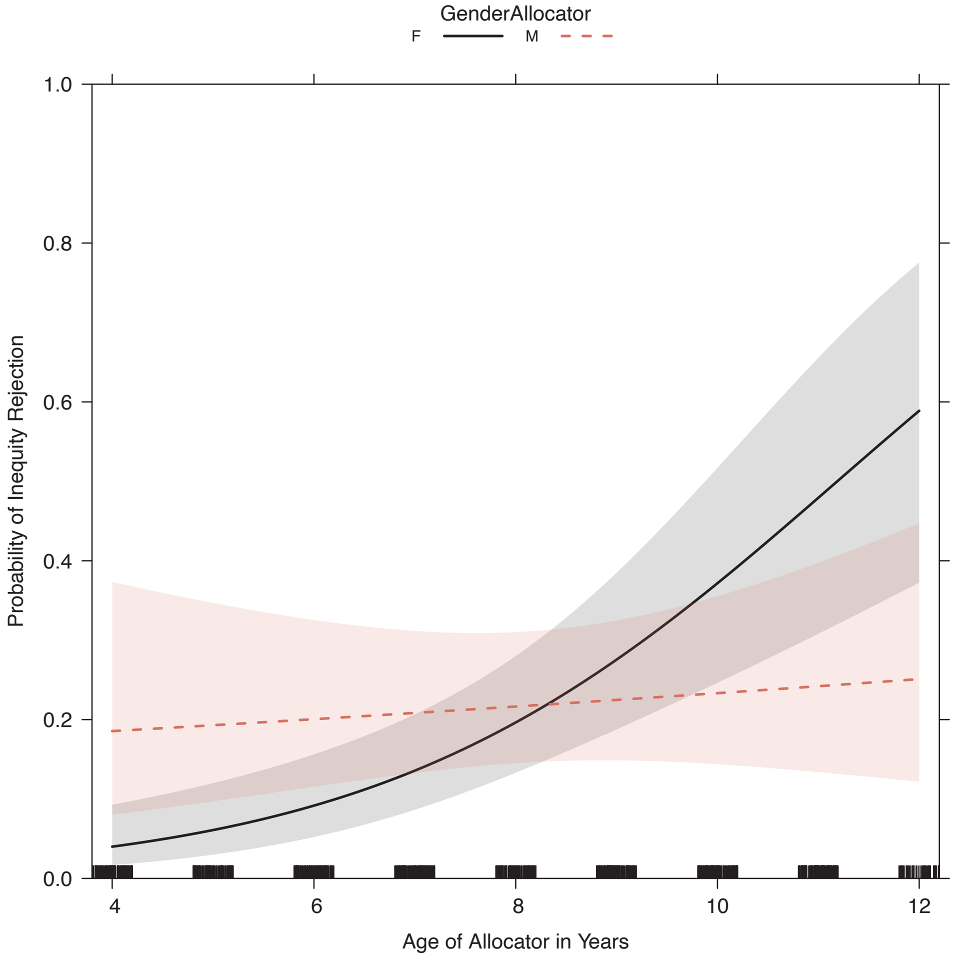

Figure S4 Predicted effects of the Age x GenderAllocator interaction for the Blake dataset. WEIRD children (Candadian + US children), children in AI condition only.

7 Sensitivity Analysis for Age-related effects

As we partitioned our complete sample in subgroups (see main manuscript) for the model-based Fehr-Schmidt analyses, the question might arise whether our experimental design was appropriately powered to detect such effects. As the method for detecting differences between groups in the Fehr-Schmidt analyses was based on a custom programmed bootstrapped permutation method (see main methods and online code), standard analyses of effect size or a power analysis cannot be readily implemented. To show the dependence of our reported effects for differences in alpha and beta parameters between age groups (main Fig. 7) on sample size, we extended the bootstrap method to also take into account the number of samples in each group, ranging from a random draw of N=10 (with a minimum of N=3 participants from each group in the permutation analysis) up until the full combined sample size for the two groups under comparison, in steps of N=5 additional participants. We repeated our bootstrap method (now with N=100 bootstraps per sample size point. The figure below depicts the 95% confidence interval on the bootstrap permutation distribution of parameter differences per sample size step, and the actual difference in parameters, for alpha (top row) and beta (bottom row), across age group comparisons (3-4 vs. 5-6 left, 5-6 vs. 7-8 middle, 3-4 vs. 7-8 right). As can be seen from the graphs, our Bonferroni-corrected significant effects (significant increase in alpha from 3-4 to 5-6; significant increase in beta from 5-6 to 7-8) were readily detectable with smaller sample sizes. In addition, the trend in the narrowing of confidence intervals suggests that with an even larger sample, the other between-age group comparisons might have reached significance as well.

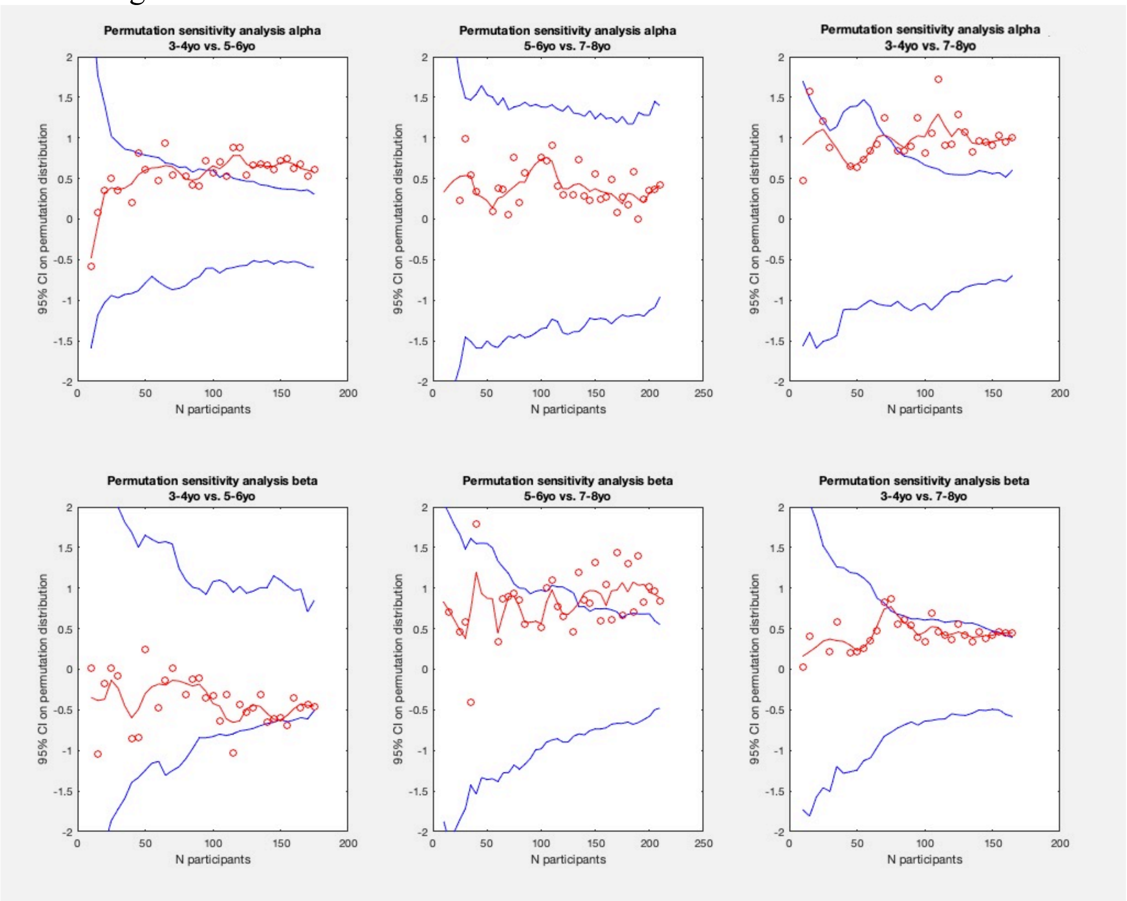

Figure S5 95% confidence intervals on the permutation distribution of between-age group differences in alpha (top row) and beta (bottom row). Blue lines indicated the smoothed (5-sample moving average). Red dots: actual difference in parameters between group for random subsample of N participants (line: 5-sample moving average).

8 Fehr-Schmidt Parameters per subgroup

The table below shows the median and standard deviation (std) across the bootstrap distribution for Fehr-Schmidt model fits. Alpha parameters are in the top part, Beta parameters in the bottom part of the table. E.g., the alpha parameter for choices made by the subgroup consisting of 5-6yo Female Allocators (paired with either Male/Female Recipients) is 0.40±0.12.

Table S12 All α- and β-value estimates of the Fehr-Schmidt model fits. F: female; M: male, FA: female allocator; MA: male allocator; FR: female recipient; MR: male recipient.

Age Group	*Alpha*	*Gender*	*Alpha*	*Dyad*	*Alpha*
3-4 year	0.10±0.04	F: allocator	0.14±0.05	FA-FR	0.02±0.03
		F: recipient	-0.03±0.04	FA-MR	0.30±0.09
		M: allocator	0.01±0.09	MA-FR	-0.27±0.13
		M: recipient	0.26±0.08	MA-MR	0.21±0.08
5-6 year	0.66±0.11	F: allocator	0.40±0.12	FA-FR	0.18±0.11
		F: recipient	0.34±0.12	FA-MR	0.76±0.16
		M: allocator	0.81±0.20	MA-FR	0.50±0.14
		M: recipient	1.00±0.21	MA-MR	1.22±0.25
7-8 year	1.08±0.14	F: allocator	1.05±0.27	FA-FR	0.64±0.05
		F: recipient	0.88±0.16	FA-MR	1.90±0.34
		M: allocator	1.08±0.25	MA-FR	1.59±0.40
		M: recipient	1.30±0.33	MA-MR	0.75±0.06
	Beta	*Gender*	*Beta*	*Dyad*	*Beta*
3-4 year	0.16±0.07	F: allocator	0.35±0.06	FA-FR	0.41±0.03
		F: recipient	0.28±0.07	FA-MR	0.24±0.12
		M: allocator	-0.22±0.21	MA-FR	-0.19±0.22
		M: recipient	0.03±0.16	MA-MR	-0.22±0.20
5-6 year	-0.23±0.16	F: allocator	-0.04±0.22	FA-FR	0.04±0.20
		F: recipient	0.05±0.19	FA-MR	-0.19±0.26
		M: allocator	-0.21±0.24	MA-FR	0.09±0.17
		M: recipient	-0.48±0.28	MA-MR	-0.62±0.31
7-8 year	0.60±0.07	F: allocator	0.77±0.22	FA-FR	0.56±0.05
		F: recipient	0.61±0.10	FA-MR	1.01±0.21
		M: allocator	0.39±0.10	MA-FR	0.64±0.17
		M: recipient	0.51±0.14	MA-MR	0.25±0.06

9 Supplemental Bibliography

1. Fox, J. Effect Displays in R for Generalised Linear Models. *J Stat Softw* **8**, 1–27 (2003).
2. Fox, J. & Weisberg, S. *An R Companion to Applied Regression*. (Sage, Thousand Oaks CA, 2019).
3. Fox, J. & Weisberg, S. Visualizing Fit and Lack of Fit in Complex Regression Models with Predictor Effect Plots and Partial Residuals. *J Stat Softw* **87**, 1–27 (2018).
4. Blake, P. R. *et al.* The ontogeny of fairness in seven societies. *Nature* **528**, 258–261 (2015).
5. Blake, P. R. *et al.* Data from: The ontogeny of fairness in seven societies. *Data Dryad* Preprint at https://doi.org/10.5061/dryad.g3925 (2016).
