# Supplementary material for: Egalitarian preferences in young children depend on the genders of the interacting partners
Source: Commun Psychol. 2024 Sep 25;2:89. doi: 10.1038/s44271-024-00139-9 (PMC11424646; doi:10.1038/s44271-024-00139-9)
Supplement: Supplementary file 3 — Reporting Summary [file 44271_2024_139_MOESM3_ESM.pdf]

nature portfolio

Corresponding author(s): Marijn van Wingerden

Last updated by author(s): Aug 21, 2024

Reporting Summary

Nature Portfolio wishes to improve the reproducibility of the work that we publish. This form provides structure for consistency and transparency in reporting. For further information on Nature Portfolio policies, see our Editorial Policies and the Editorial Policy Checklist.

Statistics

For all statistical analyses, confirm that the following items are present in the figure legend, table legend, main text, or Methods section.

n/a	Confirmed	
☐	☒	The exact sample size (*n*) for each experimental group/condition, given as a discrete number and unit of measurement
☐	☒	A statement on whether measurements were taken from distinct samples or whether the same sample was measured repeatedly
☐	☒	The statistical test(s) used AND whether they are one- or two-sided<br>*Only common tests should be described solely by name; describe more complex techniques in the Methods section.*
☐	☒	A description of all covariates tested
☐	☒	A description of any assumptions or corrections, such as tests of normality and adjustment for multiple comparisons
☐	☒	A full description of the statistical parameters including central tendency (e.g. means) or other basic estimates (e.g. regression coefficient) AND variation (e.g. standard deviation) or associated estimates of uncertainty (e.g. confidence intervals)
☒	☐	For null hypothesis testing, the test statistic (e.g. *F*, *t*, *r*) with confidence intervals, effect sizes, degrees of freedom and *P* value noted<br>*Give P values as exact values whenever suitable.*
☒	☐	For Bayesian analysis, information on the choice of priors and Markov chain Monte Carlo settings
☐	☒	For hierarchical and complex designs, identification of the appropriate level for tests and full reporting of outcomes
☐	☒	Estimates of effect sizes (e.g. Cohen's *d*, Pearson's *r*), indicating how they were calculated

Our web collection on statistics for biologists contains articles on many of the points above.

Software and code

Policy information about availability of computer code

Data collection	No software
Data analysis	Matlab R2016 (MathWorks, Natick, Massachussets, U.S.A.), IBM SPSS Statistics 22 (IBM, New York, U.S.A.), RStudio 2021.09.0, R 4.1.3, GIMP and Inkscape. R packages include:<br>• Effects 4.2-270–72<br>• Tidyverse 1.3.190<br>• lme4 1.12969<br>and data I/O packages.

For manuscripts utilizing custom algorithms or software that are central to the research but not yet described in published literature, software must be made available to editors and reviewers. We strongly encourage code deposition in a community repository (e.g. GitHub). See the Nature Portfolio guidelines for submitting code & software for further information.

Data

Policy information about availability of data

All manuscripts must include a data availability statement. This statement should provide the following information, where applicable:

- Accession codes, unique identifiers, or web links for publicly available datasets
- A description of any restrictions on data availability
- For clinical datasets or third party data, please ensure that the statement adheres to our policy

Raw data is available open access in the OSF repository for this project (https://osf.io/pk6h5/). The data from the original study by Blake et al. is available via DataDryad (https://datadryad.org/stash/dataset/doi:10.5061/dryad.g3925).

Human research participants

Policy information about studies involving human research participants and Sex and Gender in Research.

Reporting on sex and gender	Our study is about the role of gender in fairness preferences in children. In line with the Sex and Gender Equity in Research (SAGER) guidelines for the use of the terms sex and gender, we consistently use the term gender (limited to the binary terms “girl” and “boy” for individuals of female/male sex) instead of sex in our description of previous research and interpretations of our own results to reflect the fact, as we argue in our manuscript, that fairness preferences are most likely shaped by a combination of socio-economic, cultural, experiential, and genetic factors. However, most studies, including ours, rely on biological sex as a proxy for (binary) gender, and it remains an open question how fairness preferences relate to a (multidimensional) gender spectrum. As justified in our manuscript, we did not poll the participants on their socially constructed (continuous) gender identity but categorized them solely based on their (assumed) binary biological sex. Data are thus presented using biological terms (female/male participants), are described disaggregated for all combinations of female/male participants, but the interpretation of our results is phrased in terms of gender-related differences between girls and boys.
Population characteristics	We tested 332 children between three and eight years (females = 176, males = 156; mean 99 age = 71.95 months, s.e.m. = 1.05 months, range = 37 – 111 months).<br>3-4 year old females: 32<br>3-4 year old males: 33<br>5-6 year old females: 56<br>5-6 year old males: 56<br>7-8 year old females: 58<br>7-8 year old males: 44<br>For full description of the population characteristics, see table 1 in the main manuscript.
Recruitment	Data were collected in five primary schools and eight daycare facilities for children in urban, middle- to upper-middle class areas (Düsseldorf, Germany). With the consent of the school / daycare facility administration, information letters were sent to the parents of the children requesting permission for their child´s participation in the study. In these, the parents were informed about the experimental procedure, anonymization, and data storage policies. We only included children whose parents had given written consent to participate in our study.
Ethics oversight	The study was approved by the Ethics Committee for non-invasive human research of Heinrich-Heine-University, Düsseldorf.

Note that full information on the approval of the study protocol must also be provided in the manuscript.

Field-specific reporting

Please select the one below that is the best fit for your research. If you are not sure, read the appropriate sections before making your selection.

☐ Life sciences ☒ Behavioural & social sciences ☐ Ecological, evolutionary & environmental sciences

For a reference copy of the document with all sections, see nature.com/documents/nr-reporting-summary-flat.pdf

Behavioural & social sciences study design

All studies must disclose on these points even when the disclosure is negative.

Study description	We studied the role of gender in the development of fairness preferences in children. To better understand how the genders of both interaction partners influence resource allocation choices, thought to reflect egalitarian preferences during social interaction, we used an established resource allocation task that is widely utilized to measure social preferences in children (Fehr et al., 2008; Moore, 2009; House, Henrich, Brosnan & Silk, 2012). An allocator was paired with a known recipient, and in four dilemmas that forced participants to reveal their fairness preferences, the allocator decided between costly or non-costly equal outcomes vs. advantageous or non-advantageous unequal reward distributions. The study yielded quantitative data.

Research sample	We tested 332 children between three and eight years (females = 176, males = 156; mean age = 71.95 months, s.e.m. = 1.05 months, range = 37 – 111 months). Data were collected in five primary schools and eight daycare facilities for children in urban, middle- to upper-middle class areas (Düsseldorf, Germany).
Sampling strategy	A conceptually similar study used N=229 children, also in 3 age groups. (Fehr et al., 2008).
Data collection	The children were welcomed and asked if they wanted to participate. They were informed that the current study was a university project to investigate how children make decisions and distribute rewards (yellow smiley stickers) between themselves and another child by choosing one of two boxes with different distributions of stickers. It was randomly decided which child started with the IA choice task. The experimenter was always the same female person. She sat opposite the subject in the choice task and first informed the participants that they could stop the experiment any time. She explained that in each box, one side (white) contains the stickers for the other child (recipient´s name is used), whereas the other side (green) contains the stickers for the allocator (the child making the decision). The number of trials was not communicated but children were informed that they could keep the stickers subsequent to the experiment. For all four trials, the experimenter verbally informed the participant of the number of stickers for each child in each box. Before children made their decision by pointing at one of the boxes, they had to repeat the number of stickers they themselves and the other child would receive in each option. This comprehension question allowed us to evaluate whether children understood the task. After each choice, the experimenter transferred the stickers from the selected decision box to the collection boxes without any feedback and arranged the next distribution in the choice boxes. The order of distributions as well as the presentation side (left or right) of the equal distribution was counterbalanced among children. After the last decision of the first child in its role as allocator, the stickers from the collection boxes were put in envelopes. Children switched position and the second child likewise performed the decision task. Envelopes were handed over to the subjects after the second child had also finished the decision task and all stickers were collected in the envelopes. Depending on their choices, the number of stickers per child varied between 6 and 16. The experimenter was not blinded to the conditions (e.g., dilemmas), as they were laid out for the child and experimentor to see.
Timing	Data were collected in 2016 in a continuous period during the school year.
Data exclusions	Data from 1 child was lost, thirty-two children who could not answer all comprehension questions correctly and 20 children who had a distinctly positive (N=6 pairs, 12 children) or negative relationship (N=4 pairs, 8 children) with their assigned partner were excluded from data analysis.
Non-participation	No one dropped out.
Randomization	Children were allocated to groups defined by age and biological sex (self-reported; see above). All experimental conditions were within-subjects, with all subjects completing all conditions

Reporting for specific materials, systems and methods

We require information from authors about some types of materials, experimental systems and methods used in many studies. Here, indicate whether each material, system or method listed is relevant to your study. If you are not sure if a list item applies to your research, read the appropriate section before selecting a response.

Materials & experimental systems

n/a	Involved in the study
☒	☐ Antibodies
☒	☐ Eukaryotic cell lines
☒	☐ Palaeontology and archaeology
☒	☐ Animals and other organisms
☒	☐ Clinical data
☒	☐ Dual use research of concern

Methods

n/a	Involved in the study
☒	☐ ChIP-seq
☒	☐ Flow cytometry
☒	☐ MRI-based neuroimaging
